# Supplementary material for: From sequence to enzyme mechanism using multi-label machine learning
Source: BMC Bioinformatics. 2014 May 19;15:150. doi: 10.1186/1471-2105-15-150 (PMC4229970; doi:10.1186/1471-2105-15-150)
Supplement: Additional file 2 — Java code of ml2db. Additional file ml2db_code.tar.gz contains the Java source code to run the multi-label machine learning experiments and save the results to database. The code’s Javadoc is included. [file 1471-2105-15-150-S2.zip › additional file 2/ml2db/ml2db/doc/main/ml2db/weka/ArffPropsTable.html]

ArffPropsTable


JavaScript is disabled on your browser.


- Overview
- Package
- Class
- Use
- Tree
- Deprecated
- Index
- Help

- Prev Class
- Next Class

- Frames
- No Frames

- All Classes

- Summary:
- Nested |
- Field |
- Constr |
- Method

- Detail:
- Field |
- Constr |
- Method


main.ml2db.weka

## Class ArffPropsTable

- java.lang.Object
- - uk.ac.standrews.utils.main.database.Table
  - - main.ml2db.weka.ArffPropsTable

- ---

    

  ```
  public class ArffPropsTable
  extends uk.ac.standrews.utils.main.database.Table
  ```

  Class to keep the name and column types for the database table to store the
  ARFF file parameters (the data files for Weka or Mulan machine learning)

  Version:
  :   12 Jun 2010

  Author:
  :   Luna De Ferrari ldf(at)st-andrews.ac.uk

- - ### Field Summary

    Fields

    | Modifier and Type | Field and Description |
    | --- | --- |
    | `static java.lang.String` | `ARFF_ID_COLUMN` |
    | `static java.lang.String` | `TABLE_NAME` |

    - ### Fields inherited from class uk.ac.standrews.utils.main.database.Table

      `m_writer`
  - ### Constructor Summary

    Constructors

    Constructor and Description

    `ArffPropsTable(uk.ac.standrews.utils.main.database.DbManager man)`
  - ### Method Summary

    Methods

    | Modifier and Type | Method and Description |
    | --- | --- |
    | `uk.ac.standrews.utils.main.database.TableRow` | `createRow(Arff arff)` |
    | `java.lang.String` | `getArffPathFromDb(int arffRecordId)` |
    | `java.lang.String` | `getArffPropsPathFromDb(int arffRecordId)` |
    | `static java.util.Vector<uk.ac.standrews.utils.main.database.TableColumn>` | `getColumnDefinitions()` Creates the table for the ARFF file records |
    | `int` | `getIdByArffPath(java.lang.String arffPath)` Returns the first arff id in the table whose arff path matches the given string (or part of it) |
    | `int` | `getIdByArffPath(java.lang.String arffPath, java.lang.String arffPropsFilePath)` Returns the first arff id in the table whose arff path matches the given string (or part of it). |
    | `ArffProperties` | `getPropertiesFromDb(int arffRecordId)` |
    | `java.lang.String` | `getPropertyValueFromTable(java.lang.String propertyName, int id)` |
    | `int` | `writeArffRecordToDb(Arff arff)` |

    - ### Methods inherited from class uk.ac.standrews.utils.main.database.Table

      `getColumnNames, getColumns, getDbManager, getTableCreator, getTableName, getTableReader, getTableRow, getTableWriter, hasKey, initialise, initialise, name, saveRow`
    - ### Methods inherited from class java.lang.Object

      `equals, getClass, hashCode, notify, notifyAll, toString, wait, wait, wait`

- - ### Field Detail


    - #### TABLE\_NAME

      ```
      public static final java.lang.String TABLE_NAME
      ```

      See Also:
      :   Constant Field Values


    - #### ARFF\_ID\_COLUMN

      ```
      public static final java.lang.String ARFF_ID_COLUMN
      ```

      See Also:
      :   Constant Field Values
  - ### Constructor Detail


    - #### ArffPropsTable

      ```
      public ArffPropsTable(uk.ac.standrews.utils.main.database.DbManager man)
      ```
  - ### Method Detail


    - #### createRow

      ```
      public uk.ac.standrews.utils.main.database.TableRow createRow(Arff arff)
      ```


    - #### getArffPathFromDb

      ```
      public java.lang.String getArffPathFromDb(int arffRecordId)
      ```


    - #### getArffPropsPathFromDb

      ```
      public java.lang.String getArffPropsPathFromDb(int arffRecordId)
      ```


    - #### getIdByArffPath

      ```
      public int getIdByArffPath(java.lang.String arffPath)
      ```

      Returns the first arff id in the table whose arff path matches the given
      string (or part of it)

      Parameters:
      :   `arffPath` - the arff file path (or part of it)


    - #### getIdByArffPath

      ```
      public int getIdByArffPath(java.lang.String arffPath,
                        java.lang.String arffPropsFilePath)
      ```

      Returns the first arff id in the table whose arff path matches the given
      string (or part of it). If the arff record is not found, it creates a new
      arff and record using the given .props file path.

      Parameters:
      :   `arffPath` - the arff file path (or part of it)
      :   `arffPropsFilePath` - the path to the .props file to create an arff


    - #### getPropertiesFromDb

      ```
      public ArffProperties getPropertiesFromDb(int arffRecordId)
      ```


    - #### getPropertyValueFromTable

      ```
      public java.lang.String getPropertyValueFromTable(java.lang.String propertyName,
                                               int id)
      ```


    - #### writeArffRecordToDb

      ```
      public int writeArffRecordToDb(Arff arff)
      ```


    - #### getColumnDefinitions

      ```
      public static java.util.Vector<uk.ac.standrews.utils.main.database.TableColumn> getColumnDefinitions()
      ```

      Creates the table for the ARFF file records

      Returns:
      :   true if the table was created


- Overview
- Package
- Class
- Use
- Tree
- Deprecated
- Index
- Help

- Prev Class
- Next Class

- Frames
- No Frames

- All Classes

- Summary:
- Nested |
- Field |
- Constr |
- Method

- Detail:
- Field |
- Constr |
- Method
